# Recurrent Liver Abscess Caused by Mucoid-Type Klebsiella pneumoniae Successfully Treated With Hepatic Artery Antibiotic Infusion Therapy: A Case Report

**DOI:** 10.7759/cureus.79451

**Published:** 2025-02-22

**Authors:** Makoto Shinohara, Ryosuke Nakano, Naruhiko Honmyo, Hiroshi Sakai, Seiichi Shimizu, Shintaro Kuroda, Hiroyuki Tahara, Masahiro Ohira, Kentaro Ide, Keigo Chosa, Kazuo Awai, Tsuyoshi Kobayashi, Hideki Ohdan

**Affiliations:** 1 Department of Gastroenterological and Transplant Surgery, Graduate School of Biomedical and Health Science, Hiroshima University, Hiroshima, JPN; 2 Department of Diagnostic Radiology, Hiroshima University, Hiroshima, JPN

**Keywords:** angiography, hepatic artery antibiotic infusion therapy, less-invasive alternative treatment, liver abscess, mucoid-type klebsiella pneumoniae

## Abstract

Liver abscesses typically respond well to a combination of antibiotics and drainage. However, some cases can be resistant to treatment and pose a clinical challenge. Infections caused by mucoid-type *Klebsiella pneumoniae *are rare, but highly invasive, and can lead to conditions such as liver abscesses and hematogenous dissemination, resulting in systemic complications. Herein, we present the case of an 80-year-old woman with recurrent multilocular liver abscess caused by mucoid-type *K. pneumoniae* who was successfully treated with hepatic artery antibiotic infusion therapy. The patient was initially managed by percutaneous drainage and intravenous antibiotic administration, but the abscess relapsed despite multiple interventions. The patient was subsequently referred to our hospital for multidisciplinary treatment. Hepatic artery antibiotic infusion therapy using cefmetazole was chosen based on the patient's preference for a less-invasive approach. This treatment led to a marked reduction in abscess size, allowing a transition to oral antibiotics and achievement of complete resolution without the need for surgical intervention. This case highlights the effectiveness of hepatic artery antibiotic infusion as a less-invasive option for managing complex, refractory liver abscesses, providing an alternative to circumvent surgical intervention. Further research is needed to define the optimal use and indications for this approach.

## Introduction

Liver abscesses are inflammatory hepatic lesions caused by bacterial, parasitic, or fungal infections [1]. They are classified as pyogenic or amoebic depending on their causes, with pyogenic abscesses being more common in developed countries and amebic abscesses being prevalent in tropical regions [2]. Fever, abdominal pain, and nausea are the most common symptoms [3-6]. Risk factors associated with the development of liver abscesses include older age, male sex, underlying diabetes mellitus, liver cirrhosis, continuous use of proton pump inhibitors, and an immunocompromised state [7-11]. Diagnosis typically involves imaging studies such as ultrasound, computed tomography (CT), and blood tests, whereas treatment usually combines antibiotics with percutaneous or surgical drainage [6]. In patients where intravenous antibiotic administration proves ineffective, drainage is difficult, or surgical resection is not feasible, hepatic artery antibiotic infusion therapy administered via a temporary catheter is reportedly effective [12,13]. Herein, we report on a case of a difficult-to-treat liver abscess caused by mucoid-type *Klebsiella pneumoniae* that relapsed repeatedly but was successfully treated using hepatic artery antibiotic infusion therapy, thereby avoiding surgical resection.

## Case presentation

**Figure 1:**
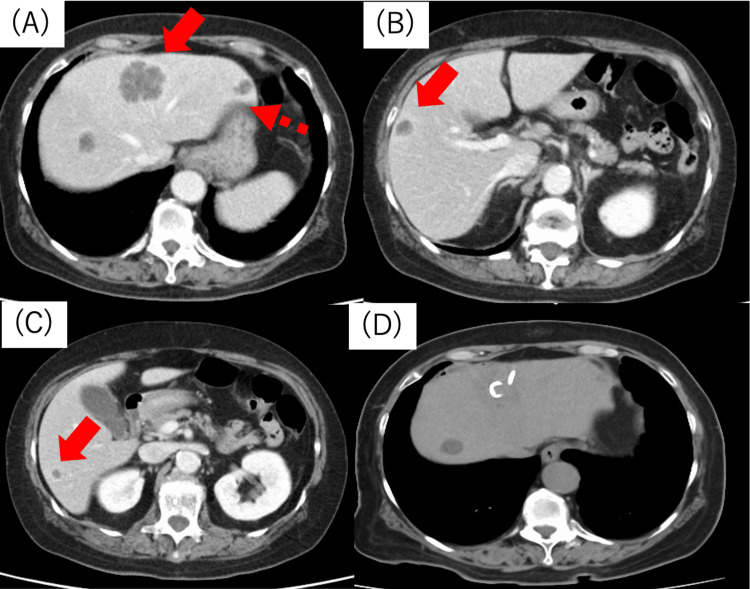
Image of a CT scan taken upon admission at the referring hospital. Contrast-enhanced CT imaging showing (A) a multifocal abscess in hepatic segment S4 (40 mm) (arrow) and an abscess in S3 (point arrow). Additional abscesses were observed in (B) S5 (arrow) and (C) S6 (arrow). (D) Percutaneous transhepatic drainage was performed to treat the multifocal abscess in hepatic S4 CT: computed tomography

An 80-year-old woman presented with a chief complaint of fever. She had a history of hypertension but not of abdominal surgery. Upon examination, her body temperature was 38°C, and she exhibited abdominal tenderness, along with epigastric discomfort. Laboratory test results revealed an elevated white blood cell count of 18,980/μL and a C-reactive protein level of 26.19 mg/dL. Mild elevations in liver enzymes were noted, with a T-bil of 1.9 mg/dL, aspartate aminotransferase level of 73 U/L, and alanine aminotransferase level of 73 U/L. Contrast-enhanced CT revealed a multilocular abscess in hepatic segment 4 (S4) of the liver and additional abscesses in S3, 5, and 6 (Figures 1A-1C). Gallstones were not observed. Abdominal magnetic resonance imaging (MRI) showed no biliary tract abnormalities. Upper and lower gastrointestinal endoscopy revealed no significant abnormalities. Percutaneous transhepatic drainage was performed for the abscess in S4 (Figure 1D), and meropenem (MEPM) (3 g/day) was administered intravenously for 27 days.

**Figure 2:**
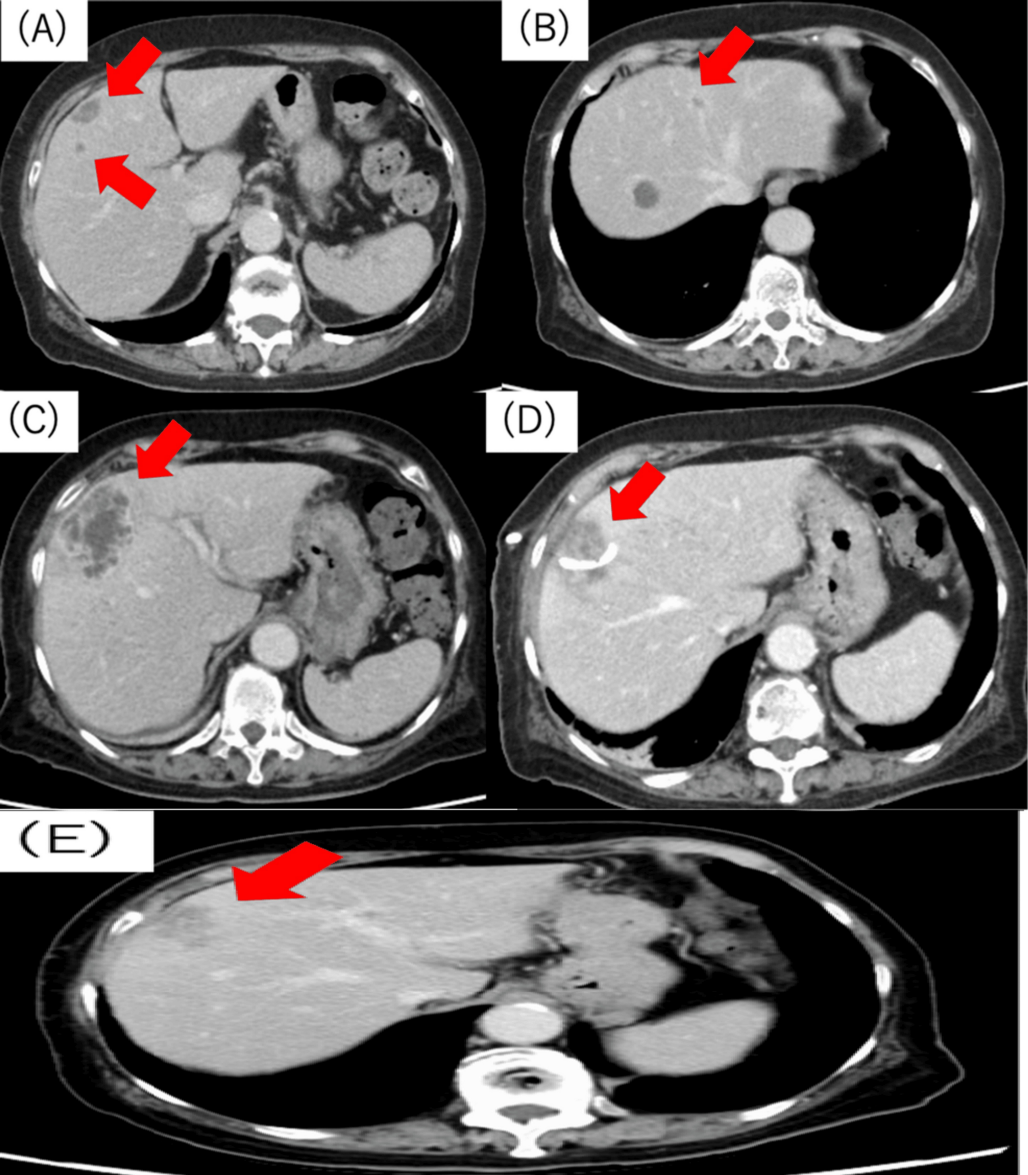
Image of a CT scan taken during readmission and at discharge from the referring hospital. Contrast-enhanced CT showing (A) abscesses in the liver S4 (20 mm) (arrow) and another in (B) S3 (arrow). (C) Antibiotic administration increased the abscess size in S4 (52 mm) (arrow). (D) Percutaneous transhepatic drainage was performed to treat the multifocal abscess in liver S4 (arrow). (E) Despite performing percutaneous transhepatic drainage and providing antibiotic treatment, the abscess remained unresolved at the time of discharge (29 mm) (arrow) CT: computed tomography

Oral levofloxacin (0.5 g/day) and metronidazole (1.5 g/day) were subsequently administered for seven days. Abscess reduction and improvement in the inflammatory response were observed, and the patient was eventually discharged. However, three months later, the patient presented with a fever and was readmitted. Contrast-enhanced CT revealed recurrent abscesses in liver segments S4 and S3 (Figures 2A, 2B). Intravenous administration of MEPM (3 g/day) was initiated, but owing to the enlargement of the S4 abscess, percutaneous transhepatic drainage of this abscess was performed (Figures 2C, 2D). Transvenous MEPM (3 g/day) was administered for seven days. Mucoid-type *K. pneumoniae *was isolated from the abscess culture. Based on the susceptibility results, the antibiotic regimen was changed to tazobactam/piperacillin (13.5 g/day) for 22 days. Although the inflammatory marker levels improved, she still experienced intermittent right upper abdominal pain. Furthermore, contrast-enhanced CT showed a residual abscess at S4 (Figure 2E).

**Figure 3:**
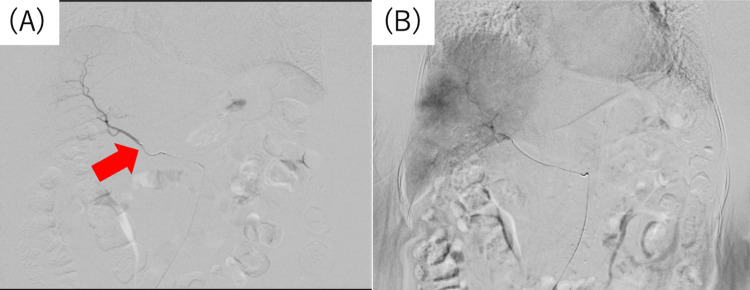
Digital subtraction angiography findings. (A) The catheter tip was placed in the right hepatic artery. (B) Digital subtraction angiography showed a faint dark staining image of hepatic S4. These findings were consistent with those of an inflammatory lesion

The patient was then referred to our hospital for multidisciplinary treatment. Although surgical resection was considered, the patient requested a less-invasive treatment, and consequently, hepatic artery antibiotic infusion therapy was planned. Under local anesthesia, the right femoral artery was punctured, and angiography was performed. The right hepatic artery originated from the superior mesenteric artery, while the left lateral segmental branch of the left hepatic artery and the left gastric artery branched from a common trunk. The A4 branch originated from the common hepatic artery. Digital subtraction angiography revealed a faint dark staining image of S4. Selective angiography revealed that the lesions in S4 were fed by both the right hepatic artery and A4 branch, but the right hepatic artery was dominant (Figure 3).

**Figure 4:**
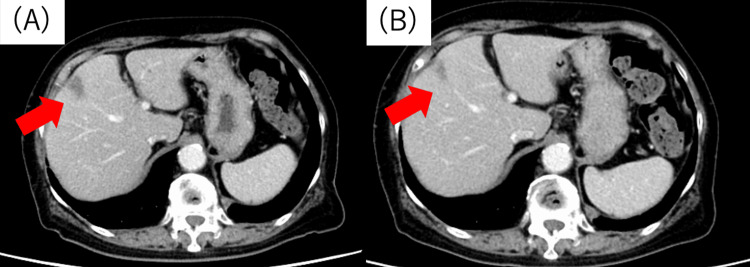
Findings of follow-up CT during hospitalization at our hospital. Contrast-enhanced CT showed that the abscess in S4 gradually shrank (arrow) (A) on day 1 (19 mm) and (B) on day 28 (13 mm) CT: computed tomography

The catheter tip was positioned in the right hepatic artery, and a port was placed subcutaneously in the lower right abdomen. Based on the findings of drug sensitivity testing, hepatic arterial infusion therapy was initiated with cefmetazole (CMZ, 4 g/day), the equivalent of that used for systemic antimicrobial therapy. Follow-up imaging evaluations were performed using contrast-enhanced CT and MRI. The abscess gradually decreased in size, and contrast-enhanced CT on day 28 showed further shrinkage (Figures 4A, 4B).

**Figure 5:**
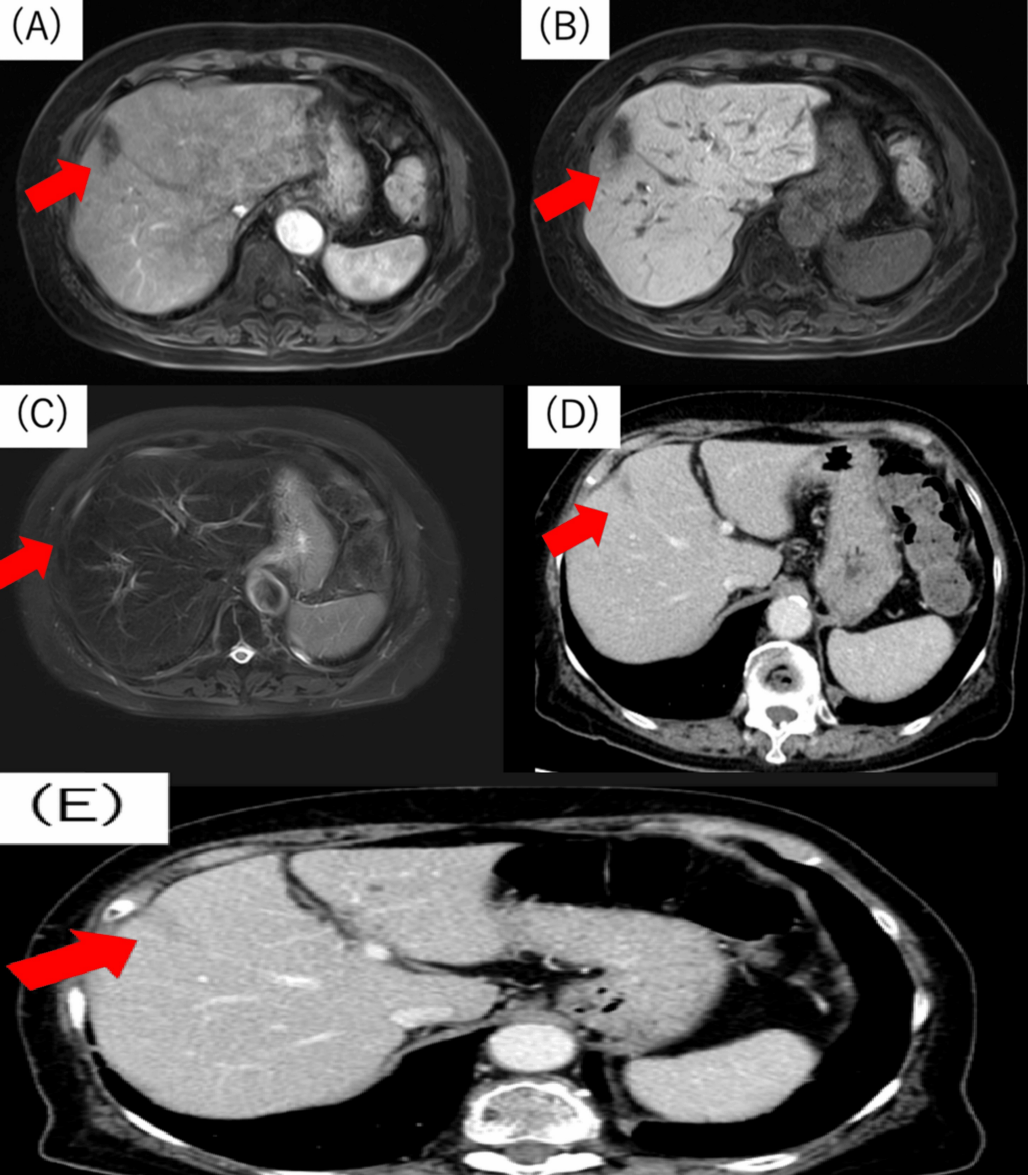
Findings from a follow-up MRI scan performed during hospitalization, CT image taken at discharge, and the outpatient follow-up CT. (A) In the arterial phase of the MRI, no evident contrast enhancement is observed (arrow). (B) In the hepatocyte phase of the MRI, a 22-mm hypointense area was observed, accompanied by a faint hypointense signal in the surrounding region (arrow). (C) In the T2-weighted imaging of the MRI, an isointense signal was observed (arrow). (D) Contrast-enhanced CT confirmed the disappearance of the abscess (arrow) (on day 51). (E) The abscess had resolved by the time of the outpatient consultation after discharge (arrow) MRI: magnetic resonance imaging; CT: computed tomography

**Figure 6:**
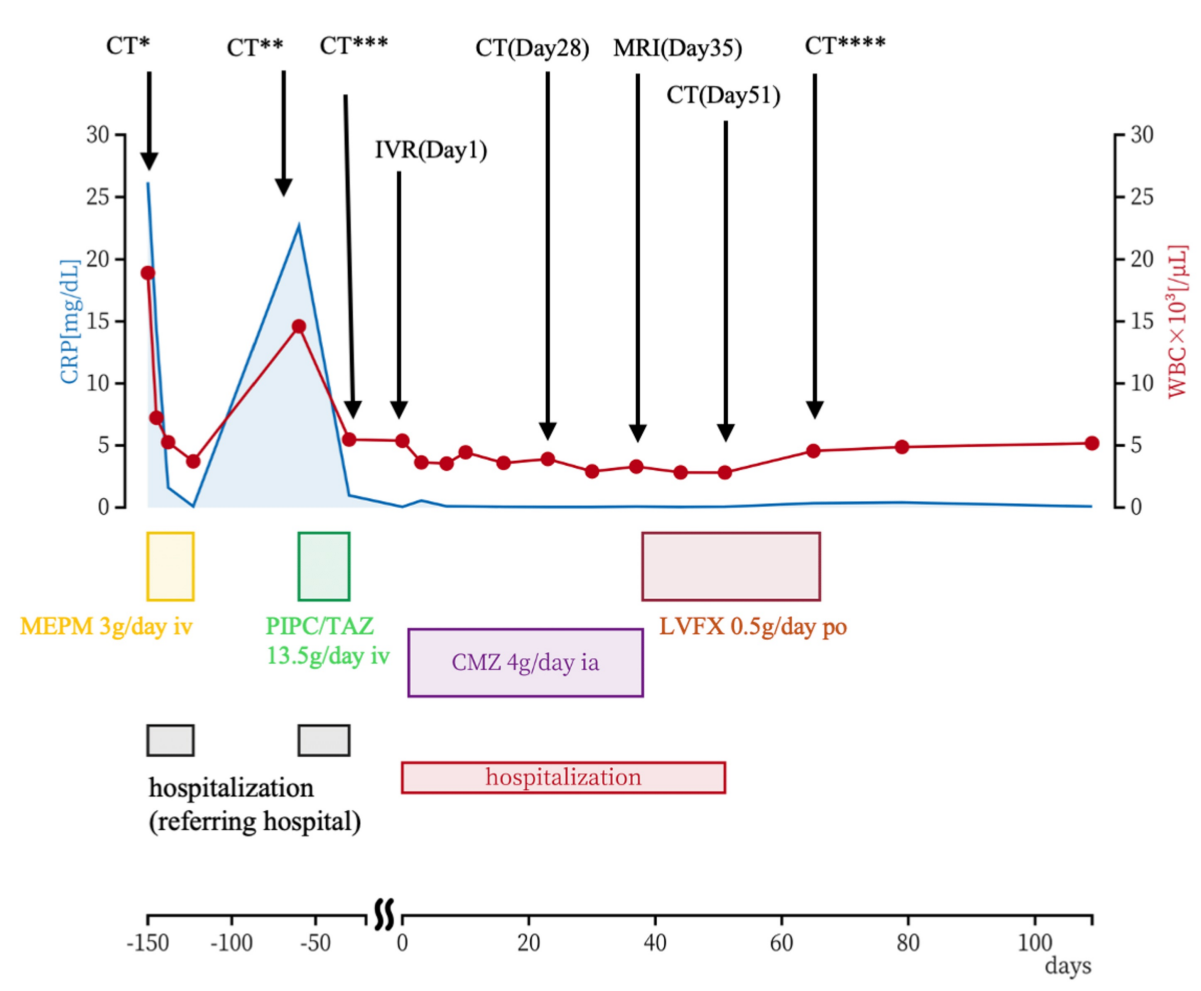
Time course of inflammatory markers and therapeutic interventions The patient’s serum CRP levels (left axis) and WBC count (right axis) are shown over a period of approximately 100 days following admission. Initial treatment with MEPM (3 g/day) is highlighted in yellow, followed by PIPC/TAZ (13.5 g/day) in green. Subsequent treatments include CMZ (4 g/day, purple) administered intra-arterially and LVFX (0.5 g/day, pink) given per os after the procedure. Hospitalization periods are marked in red, with hospitalization at the referring hospital denoted in gray. CT^*^ was performed during the patient’s initial admission at the referring hospital, CT^**^ during readmission to the referring hospital, CT^***^ at discharge from the referring hospital after readmission, and CT^****^ during an outpatient follow-up visit after discharge from our hospital CRP: C-reactive protein; WBC: white blood cell; MEPM: meropenem; LVFX: levofloxacin; CT: computed tomography; CMZ: cefmetazole; PIPC/TAZ: piperacillin/tazobactam; MRI: magnetic resonance imaging; IVR: interventional radiology

On day 35, the MRI revealed scarring from the abscess (Figures 5A-5C), and the antimicrobial therapy was switched to oral levofloxacin (500 mg/day). On day 51, contrast-enhanced CT confirmed the abscess resolution, and the patient was discharged (Figure 5D). The patient continued to be followed up as an outpatient, with no signs of liver abscess recurrence (Figures 5E, 6).

## Discussion

This case involved a liver abscess caused by mucoid-type *K. pneumoniae* that recurred repeatedly despite intravenous antibiotic administration and percutaneous transhepatic abscess drainage. We successfully treated the abscess with antibiotic infusion therapy via the hepatic artery, thereby avoiding the need for surgical liver resection.

Liver abscesses are broadly classified into bacterial and amebic types based on their causes [2]. Gram-negative bacteria, particularly *Escherichia coli* and *K. pneumoniae*, are the most common causative bacterial pathogens. In Asia, *K. pneumoniae* is the predominant cause [6,14]. Infection pathways include the biliary, portal vein, arterial, traumatic, direct extension, and idiopathic routes. Although rare, liver abscesses are of grave concern, with a reported mortality rate of 14%, according to a 2013 study by Kuo et al. involving 431 patients [15]. Standard treatment for liver abscesses involves intravenous antibiotics and drainage, and surgical resection is considered if drainage is insufficient. Early antibiotic administration is associated with low mortality and a brief hospital stay [16]. Factors contributing to intravenous antibiotic treatment resistance and drainage include multiple abscesses, abscess size >5 cm, thick pus-containing necrotic tissue [17], biliary abnormalities associated with the abscess [18], isolated multiloculated abscesses with thick capsules, and the presence of surgical diseases causing abscesses in the abdominal cavity [19]. In our patient, the abscess was multiloculated, multifocal, and separated by high-viscosity septa, which are risk factors for resistance to intravenous antibiotic treatment.

In the current case, the causative organism was mucoid-type *K. pneumoniae*. Certain cases of liver abscesses have severe clinical courses depending on the causative organism, particularly those caused by mucoid-type *K. pneumoniae*. *K. pneumoniae* is a gram-negative bacillus with a capsule; in certain serotypes, such as K1 and K2, excessive production of capsular polysaccharides results in a thick capsule; this phenotype is called the mucoid type. This type of infection is highly invasive and can cause liver abscesses and hematogenous dissemination, leading to bacteremia, meningitis, endophthalmitis, empyema, and necrotizing fasciitis, often resulting in severe systemic infections [14,20]. This phenotype is linked to plasmid-encoded virulence factors, including rmpA and the production of a substance distinct from colanic acid and capsular polysaccharides [21]. Mucoid strains exhibit increased virulence, serum resistance, and phagocytosis resistance [22]. The relationship between mucoidy, antibiotic resistance, and biofilm formation contributes to the pathogen's persistence and virulence [23,24]. In the present case, in addition to the morphological features of the liver abscess, the causative organism, mucoid-type *K. pneumoniae*, was suspected to be resistant to intravenous antimicrobial therapy, despite the administration of antibiotics to which the causative organism was confirmed to be susceptible.

Hepatic arterial infusion therapy with antibiotics is reportedly effective in cases where drainage therapy is difficult because of the location, multiloculated nature, and characteristics of the abscess or resistance to intravenous antibiotics [12,13,25]. Methods of arterial infusion of antibiotics include one-shot injection, intermittent infusion, and continuous infusion. According to Sakata et al. [25], the average time to abscess improvement is 28 days with the one-shot injection method, 18 days with a combination of percutaneous drainage and one-shot injection, and 36 days with intravenous administration alone. Matoba et al. [12] reported that the average time to abscess resolution after intermittent infusion was 10.8 days. Therefore, intermittent infusion therapy is expected to reduce treatment duration, possibly by achieving higher antibiotic concentrations within the liver and bile than those with one-shot injections or intravenous therapy [12,26]. Furthermore, standard intravenous dosing regimens were found sufficient [12]. Maintaining high local antibiotic concentrations allows more effective bacterial suppression and may be beneficial in difficult-to-treat cases. However, there is a reported case where hepatic arterial infusion therapy was ineffective, ultimately requiring surgical intervention [27]. In our patient, a hepatic arterial infusion port was placed in the right femoral artery, and CMZ (4 g/day) was administered intra-arterially for 40 days. After the confirmation of abscess scarring on CT and MRI, the antibiotic was switched to oral levofloxacin, and the patient was discharged on day 51. The patient has since been followed up as an outpatient with no recurrence of the liver abscess. Overall, intra-arterial antibiotic administration may be valuable in managing liver abscesses. We summarized five studies, including our case, that reported the use of hepatic arterial infusion therapy for liver abscesses (Table 1).

**Table 1:** Summary of five cases undergoing hepatic arterial infusion therapy for liver abscess ^*^The period of hepatic artery antibiotic infusion NA: not available

Study	No. of cases	Age	Gender	Treatment method	Treatment Duration^*^	Outcome
Peretz et al. [13]	One case	41 years	Female	Hepatic artery antibiotic infusion therapy	One month	Cure
Sakata et al. [25]	Eight cases	NA	NA	Hepatic artery antibiotic infusion therapy	One shot	Cure
		NA	NA		One shot	Cure
		NA	NA		One shot	Recurrence, ultimately cured
		NA	NA		Two shots	Cure
		NA	NA	Hepatic artery antibiotic infusion therapy plus percutaneous drainage	One shot	Cure
		NA	NA		One shot	Cure
		NA	NA		One shot	Cure
		NA	NA		Two shots	Cure
Kurosaki et al. [27]	One case	61 years	Female	Hepatic artery antibiotic infusion therapy plus percutaneous drainage	Four days	Conversion to Surgery
Matoba et al. [12]	Eight cases	48 years	Male	Hepatic artery antibiotic infusion therapy	Eight days	Cure
		76 years	Male		11 days	Cure
		56 years	Male		12 days	Cure
		65 years	Male		10 days	Death
		69 years	Female		12 days	Death
		76 years	Male		24 days	Cure
		64 years	Male		14 days	Cure
		92 years	Female		19 days	Cure
Our case	One case	80 years	Female	Hepatic artery antibiotic infusion therapy	35 days	Cure

To date, other than small splenic infarcts, no complications related to hepatic artery antibiotic infusion therapy for liver abscesses have been reported [12]. However, hepatic artery infusion therapy for liver cancer may lead to complications. Common issues include catheter (10%-26%), vascular (5%-10%), and biliary (2%-8%) complications [28]. Infectious complications, such as cholangitis, liver abscess, and septicemia, occur in 3.4% of patients [29]. Proper management and a team approach can minimize the frequency and severity of complications related to hepatic artery infusion therapy [30]. No significant complications were observed with respect to the safety of hepatic arterial infusion therapy. No bleeding occurred at the puncture site, and the patient was able to ambulate the day after the procedure. Additionally, no liver dysfunction was observed after high-dose antibiotic administration. Antibiotic infusion therapy through the hepatic artery may be a safe and effective alternative for treating liver abscesses in cases where other methods have been unsuccessful.

## Conclusions

We present a case of a refractory liver abscess that was successfully treated using hepatic antimicrobial therapy. Liver abscesses are sometimes difficult to treat using percutaneous transhepatic drainage and may be unsuitable for surgical resection. In such cases, hepatic arterial infusion therapy may be a highly effective alternative. However, only few cases have been reported and the specific indications for using this method have not yet been established, necessitating further investigation.
